# Supplementary material for: Dysregulation of PRMT5 in chronic lymphocytic leukemia promotes progression with high risk of Richter’s transformation
Source: Nat Commun. 2023 Jan 6;14:97. doi: 10.1038/s41467-022-35778-1 (PMC9823097; doi:10.1038/s41467-022-35778-1)
Supplement: Supplementary file 1 — Supplementary Information [file 41467_2022_35778_MOESM1_ESM.pdf]

SUPPLEMENTARY MATERIALS

Supplemental Figure 1

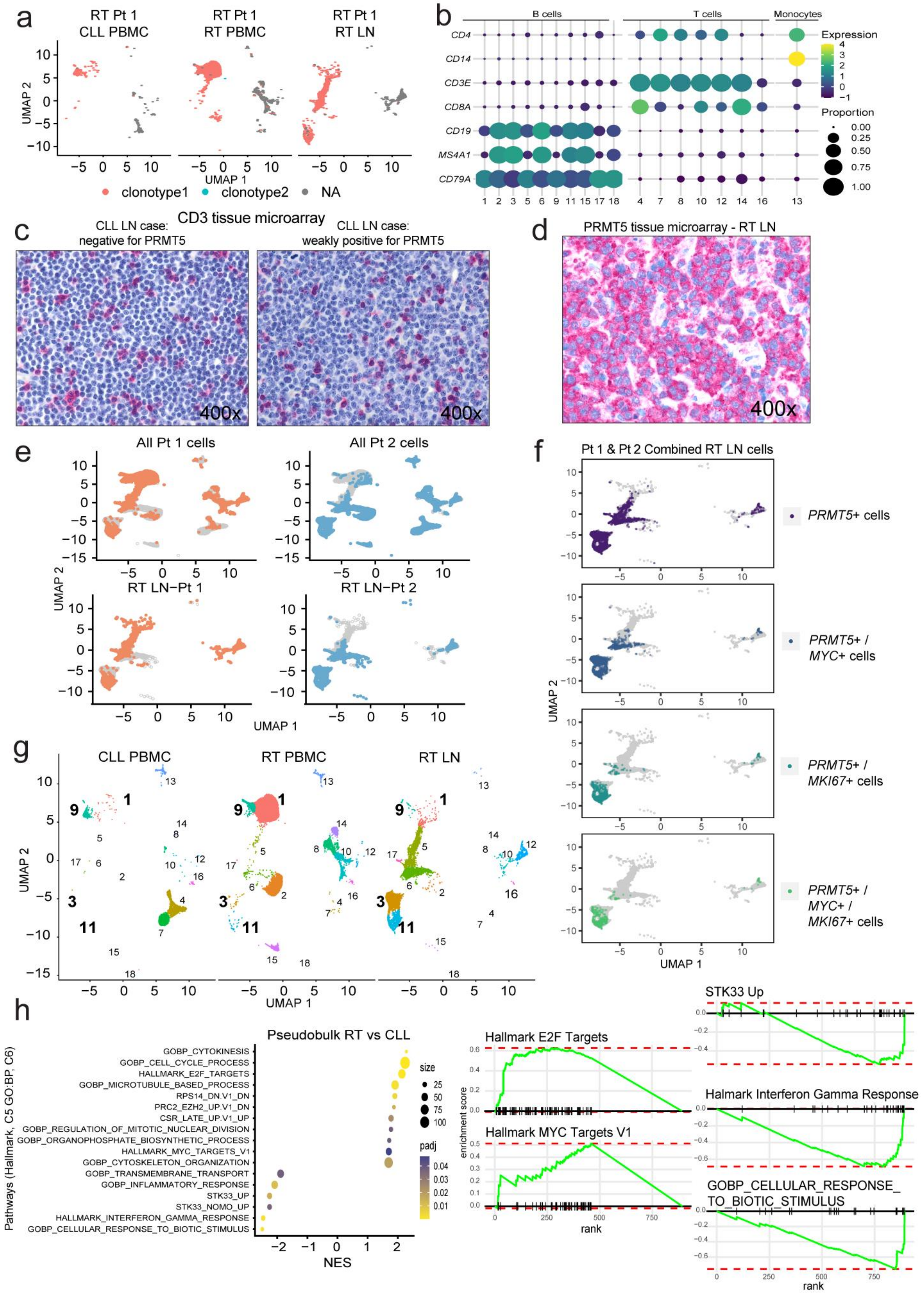

Supplemental Figure 1: Analysis of CLL-to-RT disease burden reveals expansion of clonally related cells containing tumor subsets with distinct transcript profile

A) ScV(D)J-seq analyzing the BCR clonotype profile of CLL-phase PBMCs, RT-phase PBMCs, and RT-LN cells in one RT patient. Colored dots indicate individual cells with matching VDJ sequence, demonstrating clonal evolution from CLL to RT phase.

B) Cell type assignment of leiden clusters (1-18) from scRNA-seq of 2 RT/CLL patients as B cells, T cells, or Monocytes by marker proportion and expression levels per cluster (Figure 1A). Expression color scale is shown as the average Log10-transformed expression.

C) Representative *CD3* staining by tissue microarray in lymph node biopsies from CLL LN cases negative or weakly positive for *PRMT5* (n=70). LN tumors in both cases are intermittently populated with *CD3*+ T cells with distinct cytologic morphology from small-to-medium sized tumor blasts with *PRMT5* positivity. Visualized at 400x.

D) Representative *PRMT5* staining by tissue microarray in lymph node biopsies from 15 RT cases. All 15 RT cases demonstrated strong positivity for *PRMT5*. Visualized at 400x.

E) Clustering of all cells (row 1) and RT LN cells (row 2) stratified by patient with cells highlighted (orange: Pt1, blue: Pt2) to indicate patient contribution to the clustering coordinates.

F) RT LN cells from Pt 1 and Pt 2 highlighted for cells coexpressing (UMI counts > 0) *PRMT5*, *MYC*, and *MKI67*.

G) Leiden clustering of cells from Pt 1 and Pt 2 stratified by disease/tissue with clusters distinguished by color and number. Bolded numbers highlight cell clusters with CLL (clusters 1 & 9) or RT (clusters 3 & 11) gene expression selected for pseudobulk differential expression analysis in Figure 1H.

H) Gene set enrichment analysis of pseudobulk RT vs CLL clusters from scRNA-seq on tissues from Patient 1 (CLL-phase PBMC, RT-phase PBMC, RT-LN) and Patient 2 (CLL -phase PBMC, RT-phase PBMC, RT-LN). Normalized Enrichment Scores (NES) indicate pathways that are upregulated or downregulated in RT vs CLL. Color scale indicates the adjusted p value and the size scale indicates the number of genes mapped to the indicated pathway. All pathways shown pass the cutoff padj<0.05. Right panel displays enrichment score curves for the indicated pathways. Source data are provided as a Source Data file.

Supplemental Figure 2

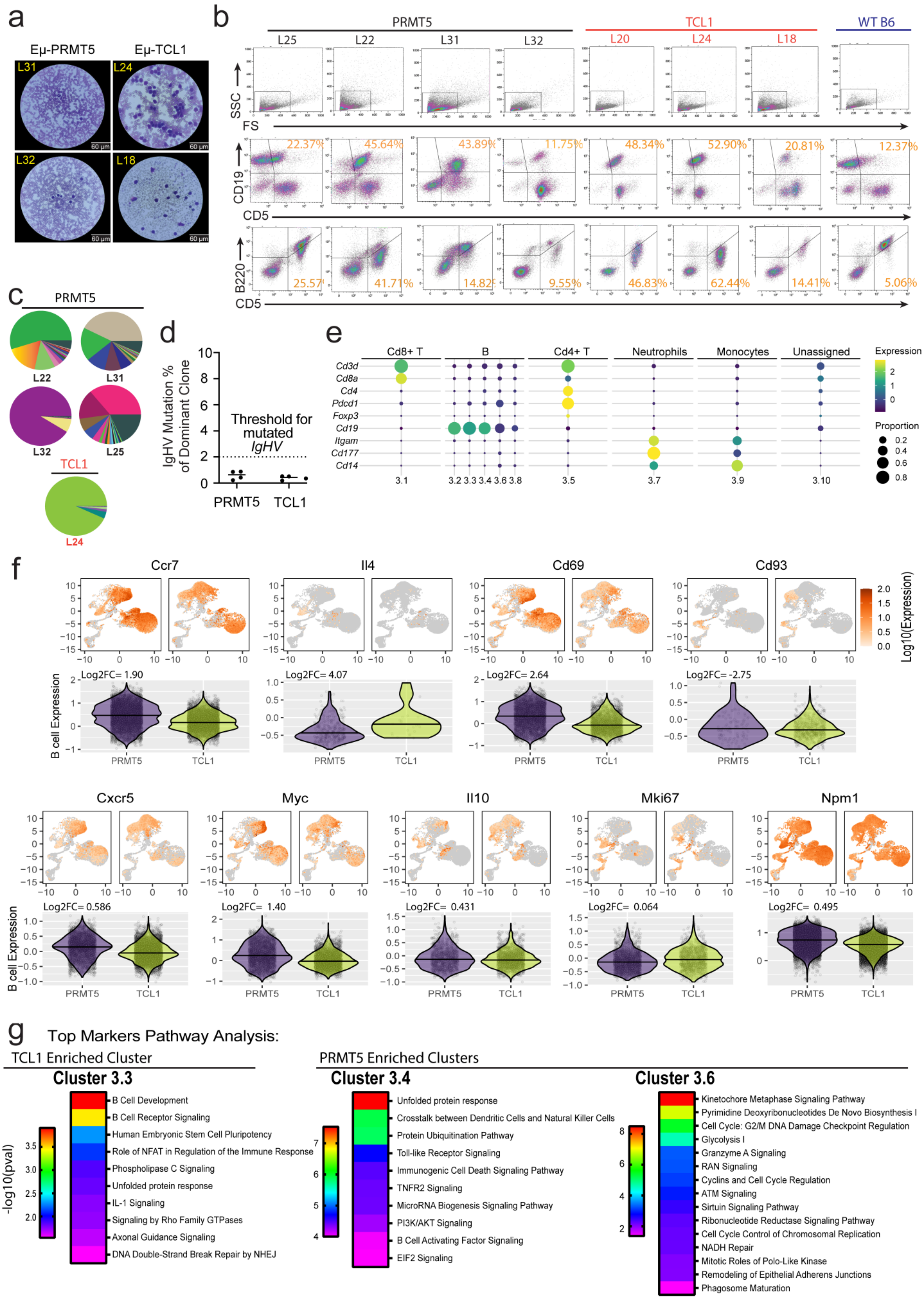

Supplemental Figure 2: CLL-like phenotype in the Eμ-PRMT5 mouse model

A) Representative peripheral blood smears from Eμ-PRMT5 (n=26) and Eμ-TCl1 (n=35) mice. Scale bar is 60 μm. May-Grünwald-Giemsa (MGG), x1,000.

B) Representative flow cytometry plots of spleen cells derived from Eμ-PRMT5 (n=5) and Eμ-TCL1 (n=3) mice at ERC. A CLL-like phenotype (*CD5*+/*CD19*+, *CD5*+/*B220*dim) is observed in Eμ-PRMT5 and Eμ-TCL1 mice, but not in WT mice.

C) CDR3 sequence abundances presented as pie-charts, reflecting clonal relation between the overall B cell populations of the spleen in Eμ-PRMT5 (n=4) and Eμ-TCL1 (n=1) mice at ERC. Top 20 most abundant CDR3 sequences are shown by mouse; shared sequences have the same color across all samples. Sequences not in the top 20 are grouped as “Others” (forest green sector).

D) Percentage of *IGVH* Mutations in the dominant clone of Eμ-PRMT5 (n=4) and Eμ-TCL1 (n=4) mice. The *IgHV* gene sequence of the dominant clone in each sample was mapped against its corresponding germline sequence. The total number of mutation events were divided by the total number of nucleotide reads to calculate the mutation percentage. *IgVH* mutation percentage below the 2% threshold is indicative of an ‘unmutated *IgHV’* classification.

E) Cell type assignment of Eμ-PRMT5 and Eμ-TCL1 spleen clusters by marker proportion and expression levels per cluster. The expression color scale is shown as the average Log10-transformed expression.

F) Visualization of variation in gene expression (Log10 size factor normalized UMI counts) of select leukemogenic genes in Eμ-PRMT5 (n=5) and Eμ-TCL1 (n=4) spleen cells visualized via UMAP. The corresponding violin plots illustrate gene expression of select leukemogenic genes in splenic B-cells, with non-zero expression, stratified by genotype and shown as the Log10-transformed expression. Log2-transformed fold changes and adjusted p values were calculated from pseudobulk differential expression analysis comparing B cell expression between models.

G) Ingenuity Pathway Analysis (IPA) of the top 50 upregulated genes in clusters Eμ-PRMT5 (3.3, 3.6) and Eμ-TCL1 (3.4) enriched clusters from the spleens of Eμ-PRMT5 (n=5) and Eμ-TCL1 (n=4) mice. Color scale reflects the range of enrichment significance [-Log2(p-value)] determined via Fisher’s exact t-test. Source data are provided as a Source Data file.

Supplemental Figure 3

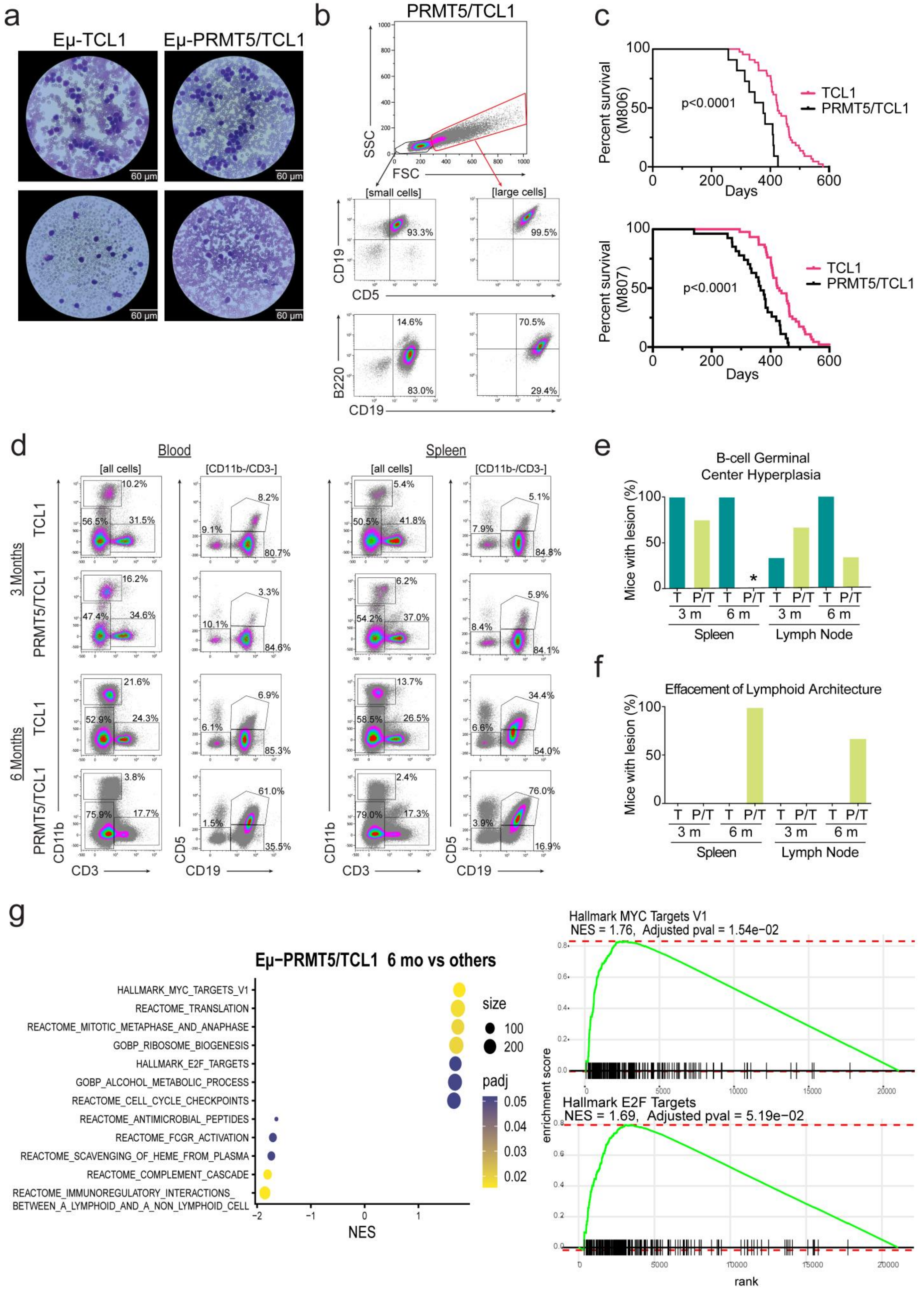

Supplemental Figure 3: Accelerated tumor kinetics and time to death in the Eμ-PRMT5/TCL1 mouse model

A) Representative peripheral blood smears from Eμ-PRMT5/TCL1 (n=78) and Eμ-TCl1 (n=36) mice demonstrate similar overall morphology. Scale bar is 60 μm. May-Grünwald-Giemsa (MGG), x1,000.

B) Representative flow cytometry plot demonstrating large circulating cells generally presenting a *Cd19+Cd5+B220*^{bright} immunophenotype.

C) Overall survival of Eμ-PRMT5/TCL1 mice derived from founder M806 (n=11) or founder M807 (n=27) and littermate Eμ-TCL1 mice (n=20 for founder M806 and 24 for founder M807). Median survival: M806, 376 days; M807, 366 days. Mantel-Cox test [M806 p=0.0001 HR=0.88 95% CI(0.46-1.7); M807 p<0.0001 HR=0.85 95% CI(0.5-1.38)].

D) Representative flow cytometry analysis of the peripheral blood and spleen from Eμ-PRMT5/TCL1 mice at 3 months (n=4) and 6 months (n=4) of age compared with Eμ-TCL1 mice at 3 months (n=3) and 6 months (n=2) of age. Staining for markers including Cd11b and CD3 indicate myeloid and T cell populations. 'CLL-like' *CD19+*/*CD5+* population is identified from [*CD11b-*/*CD3-*] population, and is more pronounced in Eμ-PRMT5/TCL1 mice at 6 months of age.

E) Bar chart showing incidence of B cell germinal center hyperplasia in the spleen and lymph node of Eμ-PRMT5/TCL1 (P/T) and Eμ-TCL1 (T) mice at 3 and 6 months of age. P/T: 3 months n=4, 6 months n=4; T mice: 3 months n=3, 6 months n=2. * indicates no hyperplasia was observed due to all analyzed mice showing progression to effacement of lymphoid architecture.

F) Bar chart showing the incidence of effacement of lymphoid architecture in spleen and lymph node of Eμ-PRMT5/TCL1 (P/T) and Eμ-TCL1 (T) mice from **E**.

G) Gene set enrichment analysis of key pathways dysregulated in 6-month-old Eμ-PRMT5/TCL1 mouse splenic B cells compared with each 3-month-old Eμ-PRMT5/TCL1 and Eμ-TCL1 mice at 3 and 6 months of age as determined via bulk RNA-sequencing. Size and color of each marker indicate the number of genes in the gene set and adjusted p-value, respectively. Source data are provided as a Source Data file.

Supplemental Figure 4

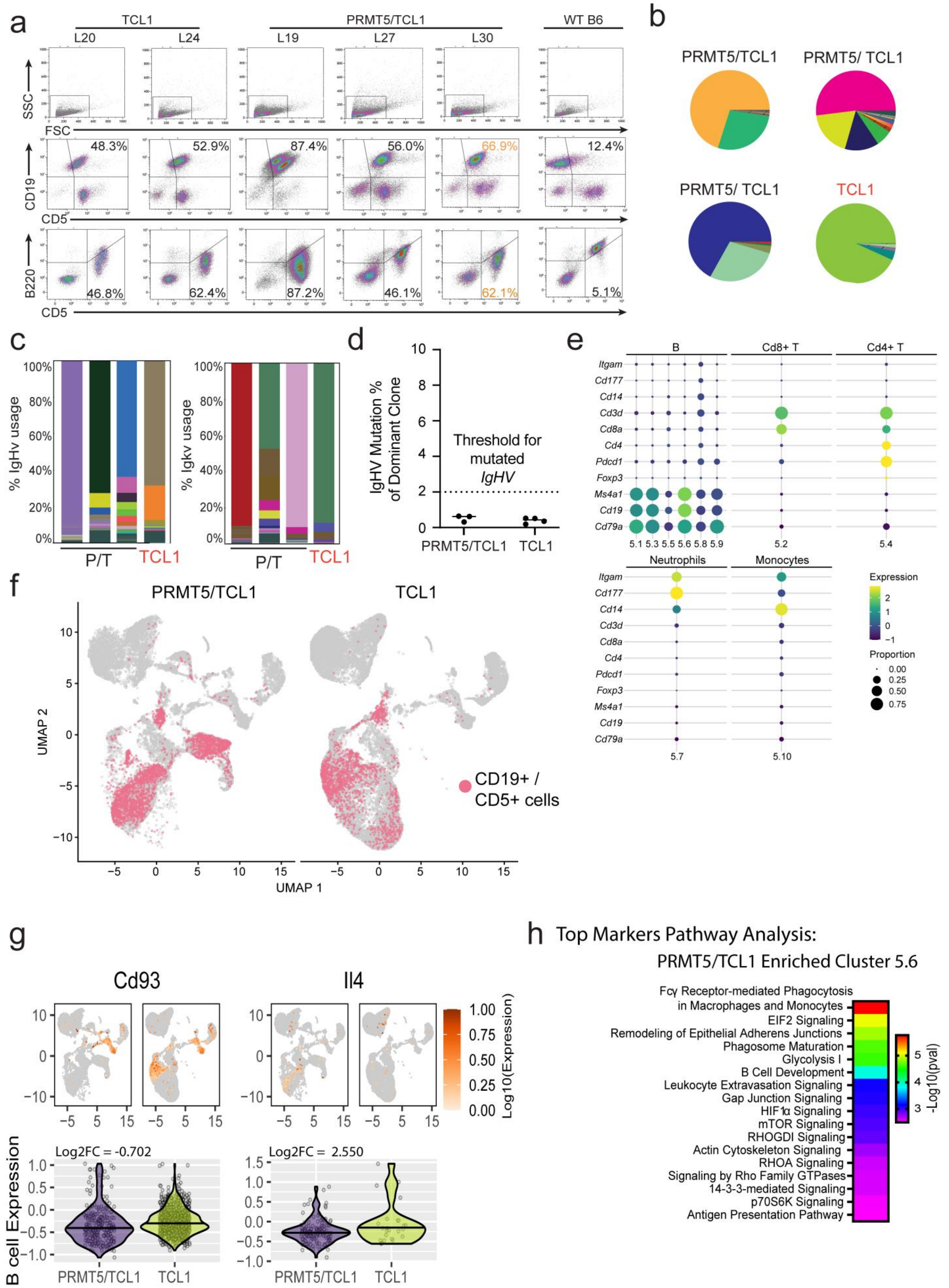

Supplemental Figure 4: CLL-like phenotype in the Eμ-PRMT5/TCL1 mouse model

A) Representative flow cytometry plots showing immunophenotypic analysis of cell populations isolated from spleens of Eμ-PRMT5/TCL1 (n=3), Eμ-TCL1 (n=2), and wild type (WT) mice upon reaching predefined removal criteria (ERC).

B) Splenic B cell CDR3 sequence abundances in Eμ-PRMT5/TCL1 (n=3) and Eμ-TCL1 (n=1) mice at ERC. Top 20 most abundant CDR3 sequences are shown by mouse; shared sequences have the same color across all samples. Sequences not in the top 20 are grouped as "Others" (<2.2% for all pie-charts, forest green).

C) B cell receptor variable gene usage in splenic CLL-like cells from Eμ-PRMT5/TCL1 and Eμ-TCL1 mice from **B** evaluated via RNA-seq. Colors indicate BCR gene usage.

D) Percentage of *IGVH* Mutations in the dominant clone from Eμ-PRMT5/TCL1 (n=3) and Eμ-TCL1 (n=4) mice. The *IGHV* gene sequence of the dominant clone in each sample was mapped against its corresponding germline sequence. The total number of mutation events were divided by the total number of nucleotide reads to calculate the mutation percentage. *IgVH* mutation percentage below the 2% threshold is indicative of an 'unmutated *IgHV'* classification.

E) Cell type assignment of Eμ-PRMT5/TCL1 and Eμ-TCL1 spleen clusters by marker proportion and expression levels per cluster. The expression color scale is shown as the average Log10-transformed expression.

F) UMAP projection of scRNA-seq analysis representing gene expression in Eμ-PRMT5/TCL1 (n=4) and Eμ-TCL1 (n=4) mouse spleen cells. Pink cells indicate 'CLL-like" cells with co-expression of Cd19+ and Cd5+ (UMI counts > 0).

G) UMAP projection of scRNA-seq analysis representing gene expression in Eμ-PRMT5/TCL1 (n=4) and Eμ-TCL1 (n=4) mouse spleen cells. Variation in *Cd93* and *Il-4* expression is shown. The corresponding violin plots illustrate gene expression in splenic B-cells, with non-zero expression, stratified by genotype and shown as the Log10-transformed expression. Log2-transformed fold changes and adjusted p values were calculated from pseudobulk differential expression analysis comparing B cell expression between models.

H) Ingenuity pathway analysis of the top 50 enriched genes in cluster 5.6 from K-means clustering of Eμ-PRMT5/TCL1 and Eμ-TCL1 spleen cells. Color scale reflects the range of enrichment significance [-Log2(p-value)] determined via Fisher's exact t-test. Source data are provided as a Source Data file.

Supplemental Figure 5

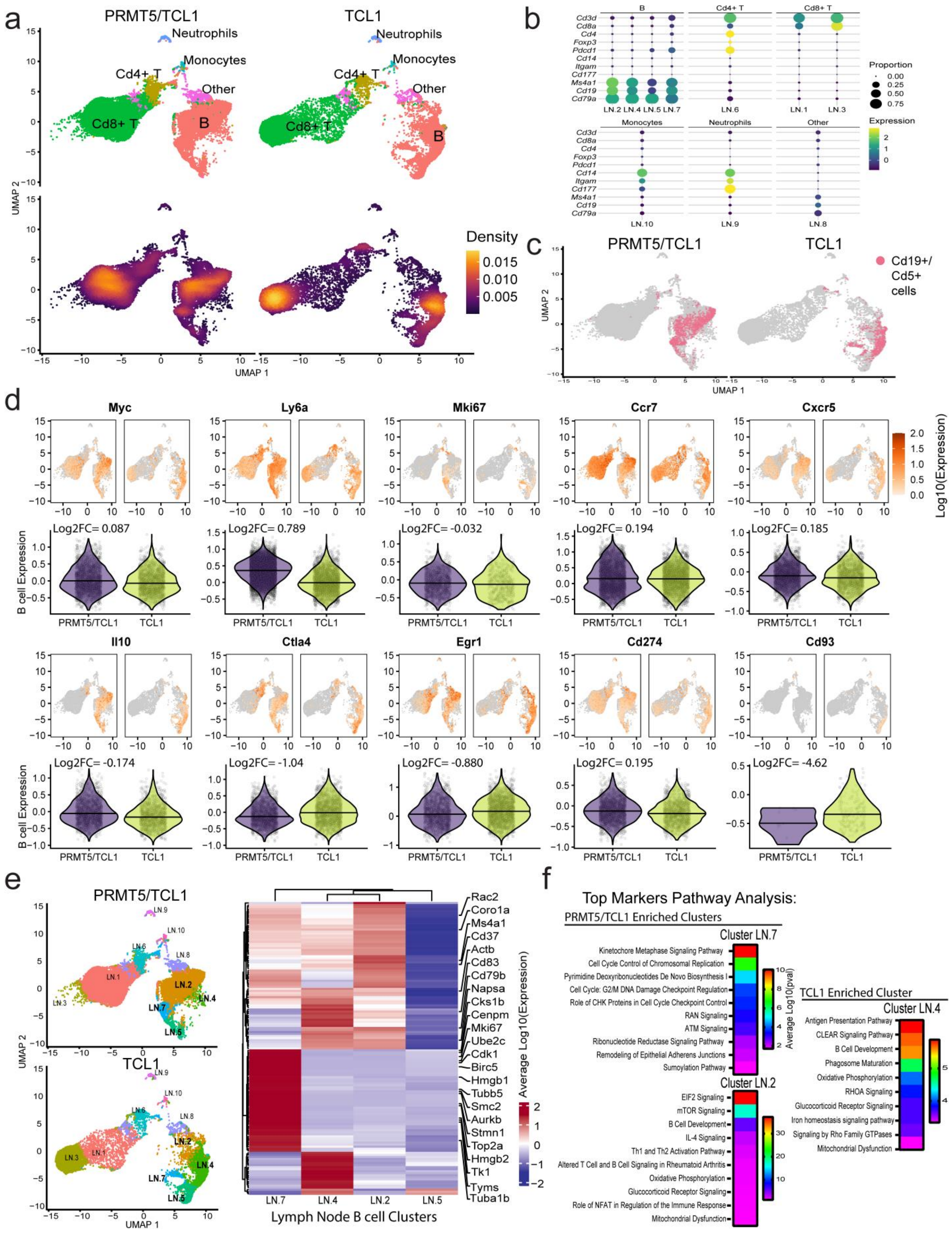

Supplemental Figure 5: scRNA-seq reveals differential tumor transcriptome in lymph node tissue from Eμ-PRMT5/TCL1 and Eμ-TCL1 mouse models

A) ScRNA-seq analysis of lymph node cells in Eμ-PRMT5/TCL1 (n=4) and Eμ-TCL1 (n=3) mice, visualized via UMAP and clustered according to K-means (n=10). B cell (*Cd19*+, *Ms4a1*+, *Cd79a*+), T cell (*Cd3*+, *Cd4*+, *Cd8*+), Mono: Monocyte (*Cd14*+, *Itgam*+), and Neutrophil (*Cd177*+, *Itgam*+) clusters were assigned as indicated in **B**. Cell density is calculated as the 2d kernel density estimate mapped to color scale.

B) Cell type assignment of Eμ-PRMT5/TCL1 and Eμ-TCL1 lymph node clusters by marker proportion and expression levels per cluster. The expression color scale is shown as the average Log10-transformed expression.

C) UMAP projection of scRNA-seq analysis representing gene expression in Eμ-PRMT5/TCL1 (n=4) and Eμ-TCL1 (n=3) mouse lymph node cells. Pink cells indicate 'CLL-like" cells with co-expression of *Cd19*+ and *Cd5*+ (UMI counts > 0 for *Cd19* and *Cd5*).

D) Visualization of variation in gene expression (Log10 size factor normalized UMI counts) of select leukemogenic genes in Eμ-PRMT5/TCL1 and Eμ-TCL1 lymph node B cells from mice in **A** visualized via UMAP. The corresponding violin plots illustrate gene expression in nodal B-cells, with non-zero expression, stratified by genotype and shown as the Log10-transformed expression. Log2-transformed fold changes and adjusted adjusted p values were calculated from pseudobulk differential expression analysis comparing B cell expression between models.

E) Heatmaps depicting the top 50 cluster specific genes expressed in lymph node B cell clusters enriched in Eμ-PRMT5/TCL1 (LN.2, LN.7) and Eμ-TCL1 (LN.4) mice. Gene expression across 143 genes is shown as the average Log10-transformed expression in nodal B cell clusters. Genes associated with leukemia and lymphoma are highlighted.

F) Ingenuity pathway analysis of the top 50 cluster specific genes in Eμ-PRMT5/TCL1 (LN.2, LN.7) and Eμ-TCL1 (LN.4) enriched lymph node clusters. Color scale reflects the range of enrichment significance [-Log2(p-value)] determined via Fisher's exact t-test. Source data are provided as a Source Data file.

Supplemental Figure 6

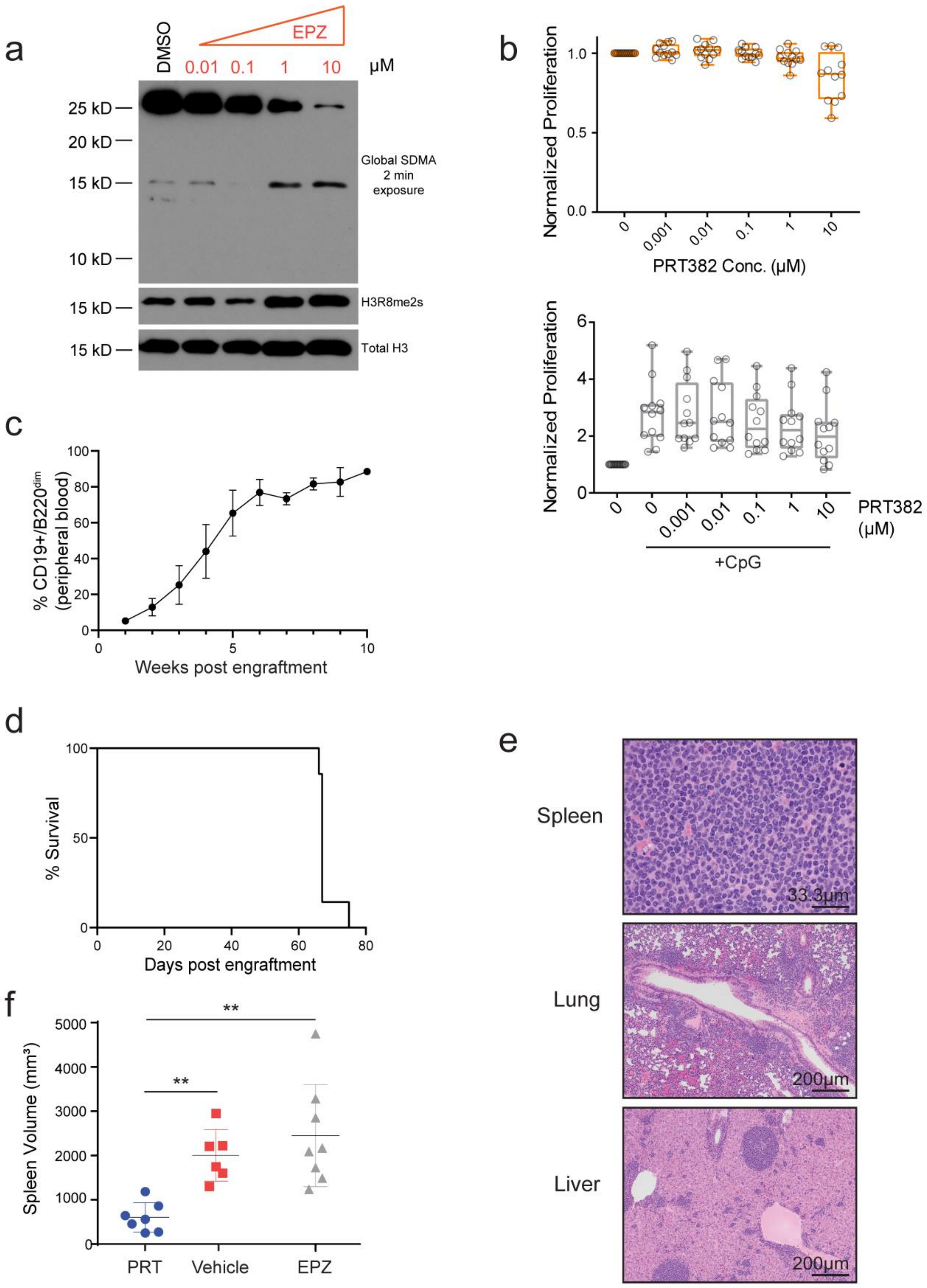

Supplemental Figure 6: *In vitro* and *in vivo* efficacy of targeted PRMT5 inhibition in CLL and CLL-like cells

A) Representative immunoblot (n=3) demonstrating dose dependent global loss of symmetric methylation dimethyl arginine residues (SDMA) in HG3 CLL cells treated with EPZ015666 for 72hr.

B) Dose-dependent decrease in proliferation, measured via MTS assay, for primary *IGVH*-U CLL cells (n=12 patients) upon 72 hr PRMT5 inhibition with increasing doses of PRT382 with and without TLR9 stimulation using 21.7 µg/mL synthetic CpG oligodeoxynucleotide. Indicated doses of PRT382 were given to cells once at initiation of this study. Circles represent individual patients. Boxplot elements reflect 25th–75th percentiles, center line reflects the median value, and whiskers reflect minimum–maximum values.

C) Viable $Cd19^{+}Cd5^{+}$ cells (5×10^{6}) from the spleen of an Eµ-PRMT5/TCL1 transgenic mouse with active CLL-like leukemia (>80% circulating in peripheral blood) and palpable splenomegaly were engrafted by tail vein into immunocompetent C57/BL6J mice. Engrafted mice were followed weekly for development of disease burden in the peripheral blood using weekly flow cytometry for percentage of $Cd5^{+}Cd19^{+}B220^{dim}$ cells. Progression of $Cd19^{+}B220^{dim}$ cells in recipient mice is plotted ±SD.

D) Engrafted mice were followed for overall survival measured by days post engraftment and visualized via Kaplan-Meier plot. Left untreated, engrafted mice succumbed to the expanding leukemia/lymphoma with a median overall survival of 67 days (range 66-75 days).

E) Representative tumor H&E histology evaluation in spleen, lung, and liver of mice engrafted with Eµ-PRMT5/Eµ-TCL1 splenic CLL-like cells (n=6). Tumor histology was similar to that of the transgenic Eµ-PRMT5/TCL1 mice. Tissues were collected upon reaching predefined removal criteria. All photographs were taken using an Olympus SC30 camera with an Olympus BX53 microscope. Scale bars as indicated.

F) Engrafted mice from (**C**) were randomly assigned to treatment conditions: PRT382 10 mg/kg, EPZ015666 50 mg/kg, or vehicle (0.5% methylcellulose, 0.1% tween) at 1 week post engraftment. All treatments were administered by oral gavage 4 contiguous days per week (Mon-Thurs). Splenic weight (g) was measured at the time mice met criteria for euthanasia. Bars indicate average ±SD for each treatment group. PRT382 vs vehicle p=0.0062; PRT382 vs EPZ015666 p=0.0077 (2-tailed paired t test). Source data are provided as a Source Data file.

Supplemental Figure 7

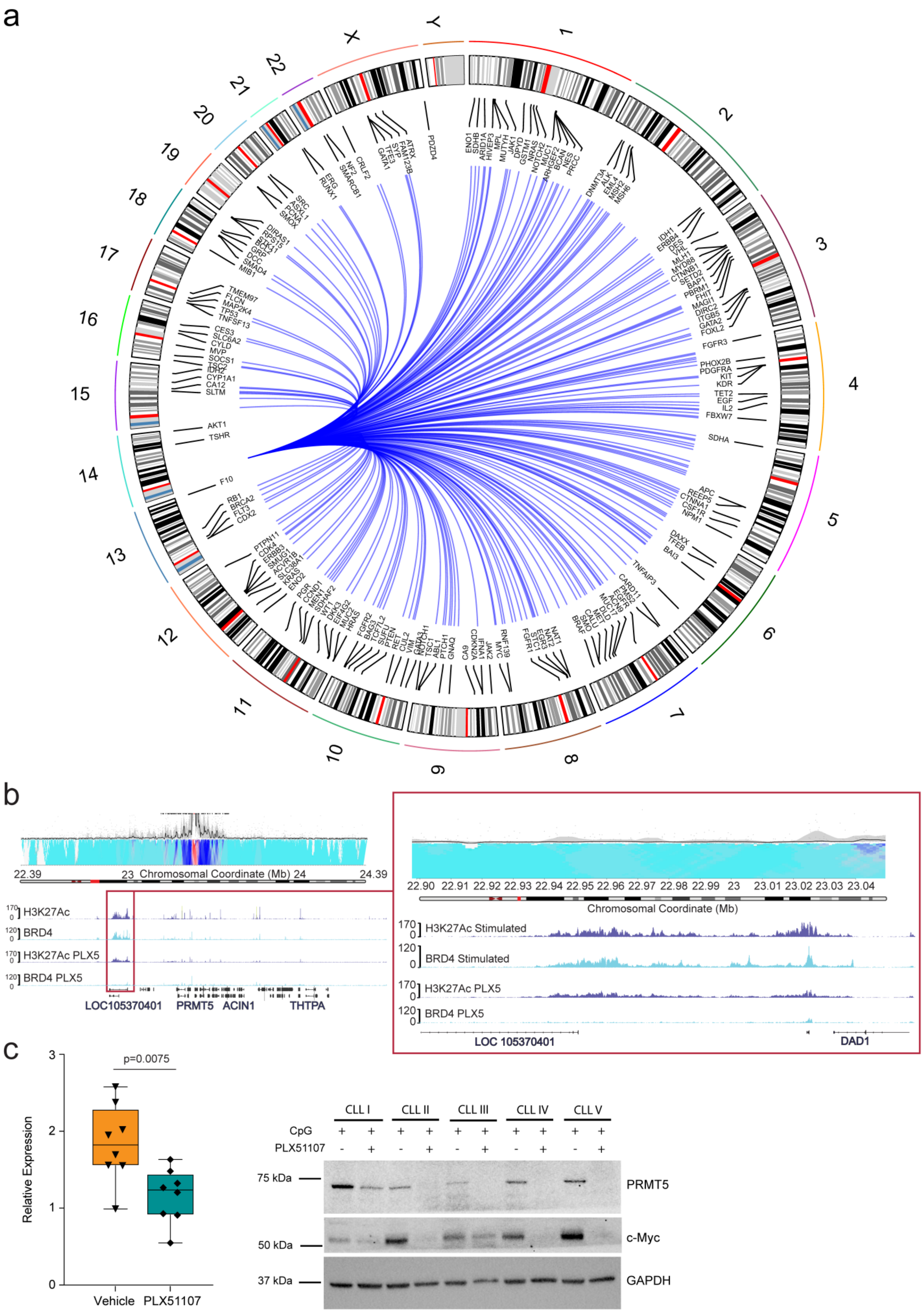

Supplemental Figure 7: BRD4 occupying promoter region reveals role in PRMT5 expression

A) Circos diagram representing trans interactions with the PRMT5 promoter region identified via 4C-seq in the HG3 CLL cell line.

B) Chromatin interactions anchored at the PRMT5 locus (shaded red) determined by 4C-seq. The 4C-seq profile (RPM normalized) shows promoter interactions with multiple regions around the PRMT5 locus (black lines). Below, ChIP-seq analysis demonstrating H3K27Ac and BRD4 binding to this locus upon vehicle, CpG, or PLX51107 treatment (red box). Right, expanded representation of ChIP-seq binding of H3K27Ac and BRD4 in all treatment groups. BRD4 occupancy is lost upon PLX51107 treatment.

C) Reduced PRMT5 mRNA (n=8 patients) and protein expression (n=5 patients) in CLL cells upon 72h 10µM PLX51107 treatment. CLL cells were stimulated with 21.7 µg/mL synthetic CpG. mRNA expression visualized as normalized expression ($2^{-\Delta\Delta CT}$) in box & whisker plot Boxplot elements reflect 25th–75th percentiles, center line reflects the median value, whiskers reflect minimum–maximum values, 2-tailed unpaired t test). Source data are provided as a Source Data file.

Supplemental Figure 8

a

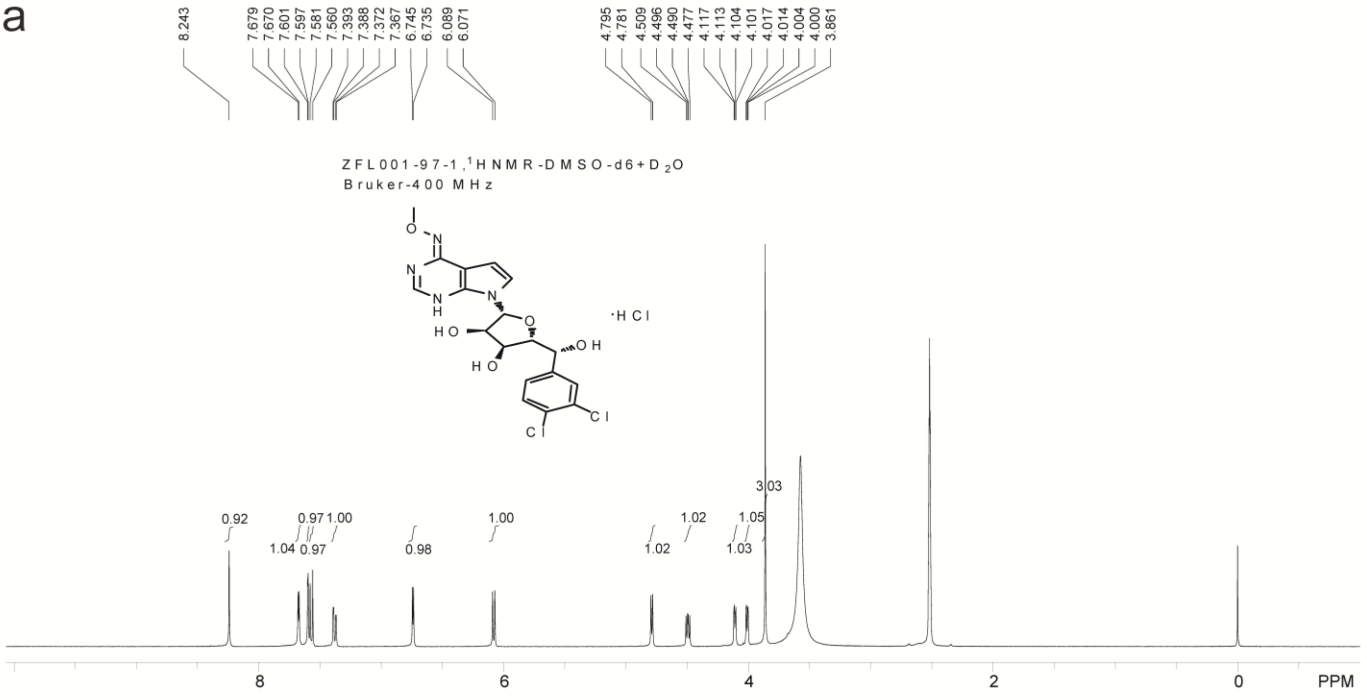

Supplemental Figure 8: ^{1}H NMR spectrum for compound PRT382

A) HNMR: 1H NMR (400 MHz, DMSO-d6) δ 8.13 (s, 1H), 7.68 – 7.53 (m, 3H), 7.38 (dd, J = 8.3, 2.0 Hz, 1H), 6.67 (s, 1H), 6.04 (d, J = 7.6 Hz, 1H), 4.79 (d, J = 5.5 Hz, 1H), 4.49 (dd, J = 7.6, 5.0 Hz, 1H), 4.08 (dd, J = 5.1, 1.5 Hz, 1H), 3.97 (dd, J = 5.4, 1.4 Hz, 1H), 3.82 (s, 3H). 1HNMR was carried out at ambient temperature.

Supplemental Figure 9

a

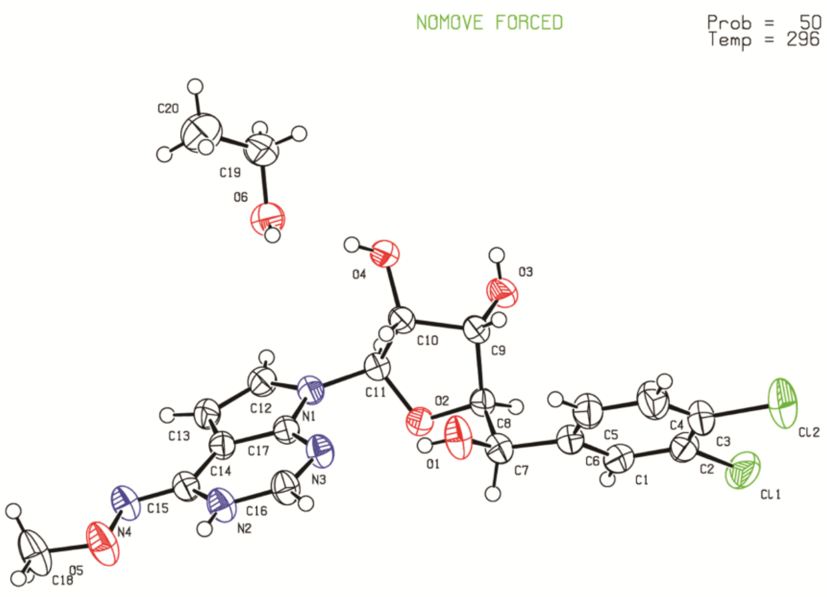

b

Crystal data for PRT382

**Compound ID**	PRT382
**Temperature**	296
**Formula**<br>***Mr***	C18H18Cl2N4O5, C2H6O1<br>*487.33*
**Crystal System**	monoclinic
**Space group**	C2
***a, b, c* (Å)**	25.6495 (5)<br>6.9352 (1)<br>15.3927 (3)
***β* (°)**	125.540 (1)
***V* (Å^3)**	2228.04 (7)
***Dc* (g cm^{-3})**	1.453
***μ* (mm^{-1})**	3.018
***R$_1$* (I>2σ(I))**	0.0297
***wR$_2$* (all data)**	0.0911
S	1.016

c

checkCIF/PLATON report PRT382

```
Bond precision:   C-C = 0.0036 A          Wavelength=1.54178
Cell:              a=25.6495(5)     b=6.9352(1)          c=15.3927(3)
                   alpha=90         beta=125.540(1)      gamma=90
Temperature:      296 K
                   Calculated                   Reported
Volume             2228.04(7)                   2228.04(7)
Space group        C 2                          C 2
Hall group         C 2y                         C 2y
Moiety formula   C18 H18 Cl2 N4 O5, C2 H6 O    C18 H18 Cl2 N4 O5, C2 H6
                                                O1
Sum formula       C20 H24 Cl2 N4 O6            C20 H24 Cl2 N4 O6
Mr                 487.33                       487.33
Dx,g cm-3          1.453                        1.453
Z                  4                            4
Mu (mm-1)          3.018                        3.018
F000               1016.0                       1016.0
F000'              1021.90
h,k,lmax           32,8,19                      32,8,19
Nref               4617[ 2508]                  4554
Tmin,Tmax          0.484,0.648                  0.553,0.754
Tmin'              0.244

Correction method= # Reported T Limits: Tmin=0.553 Tmax=0.754
AbsCorr = MULTI-SCAN
Data completeness= 1.82/0.99        Theta(max)= 75.351
R(reflections)= 0.0297( 4447)       wR2(reflections)= 0.0911( 4554)
S = 1.016                   Npar= 307
```

The following ALERTS were generated.

Alert level C

```
PLAT911_ALERT_3_C Missing # FCF Refl Between THmin & STh/L=  0.600        8 Report
```

Alert level G

```
PLAT128_ALERT_4_G Alternate Setting for Input Space Group  C2            I2 Note
PLAT395_ALERT_2_G Deviating  X-O-Y  Angle from 120 Deg for  O5        108.9 Degree
PLAT398_ALERT_2_G Deviating  C-O-C  Angle from 120 Deg for O2         110.0 Degree
PLAT791_ALERT_4_G The Model has Chirality at C7       (Chiral SPGR)       R Verify
PLAT791_ALERT_4_G The Model has Chirality at C8       (Chiral SPGR)       R Verify
PLAT791_ALERT_4_G The Model has Chirality at C9       (Chiral SPGR)       S Verify
PLAT791_ALERT_4_G The Model has Chirality at C10      (Chiral SPGR)       R Verify
PLAT791_ALERT_4_G The Model has Chirality at C11      (Chiral SPGR)       R Verify
PLAT912_ALERT_4_G Missing # of FCF Reflections Above STh/L=  0.600        9 Note
PLAT913_ALERT_3_G Missing # of Very Strong Reflections in FCF ....        2 Note
PLAT933_ALERT_2_G Number of OMIT Records in Embedded .res File ...        9 Note
PLAT978_ALERT_2_G Number C-C Bonds with Positive Residual Density.        7 Note
```

```
 0 ALERT level A    = Most likely a serious problem - resolve or explain
 0 ALERT level B    = A potentially serious problem, consider carefully
 1 ALERT level C    = Check. Ensure it is not caused by an omission or oversight
12 ALERT level G    = General information/check it is not something unexpected
```

Supplemental Figure 9: Structure of PRT382 elucidated from single crystal x-ray data

A) Chirality for carbons as shown. Atoms indicated by color: C, black; O, blue; Cl, green.

B) Tabulated crystal data for compound PRT382.

C) CheckCIF report for compound PRT382.

Supplemental Figure 10

a

Waters LCMS02 Report	Vial:2.5	Time:10:25:24<br>Injection Volume:1.0000	MP:C:0.1%FA/H2O D:0.1%FA/ACN Col.:BEH C18:<br>Inlet Method:C80D20

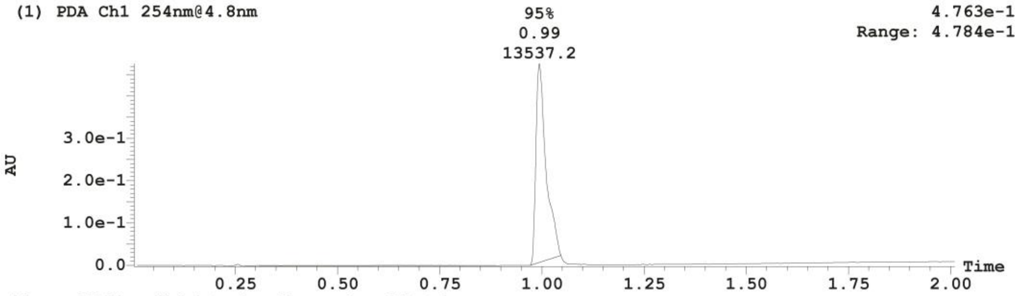

Time	Width	Height	AreaAbs	Area %Total
0.62	0.622	2e+003	5342e+002	5.27
0.99	0.075	5e+005	3537e+004	94.73

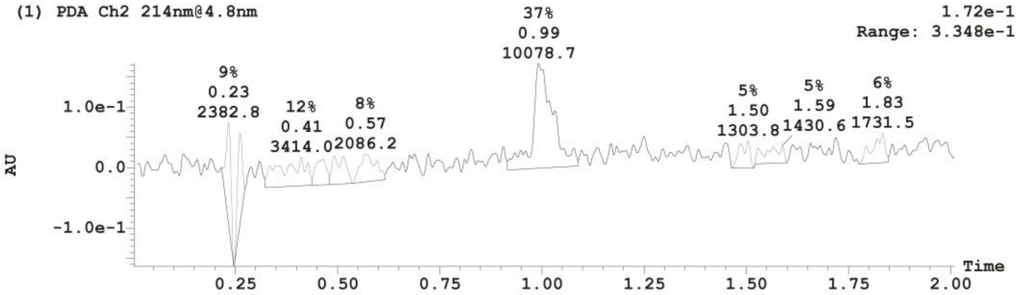

Time	Width	Height	AreaAbs	Area %Total
0.23	0.030	2e+005	3828e+003	8.67
0.26	0.025	1e+005	0299e+003	7.38
0.41	0.115	5e+004	4140e+003	12.42
0.47	0.042	4e+004	3491e+003	4.91
0.49	0.057	5e+004	3910e+003	6.15
0.57	0.080	5e+004	0862e+003	7.59
0.99	0.173	2e+005	0079e+004	36.65
1.50	0.052	5e+004	3038e+003	4.74
1.59	0.078	3e+004	4306e+003	5.20
1.83	0.072	5e+004	7315e+003	6.30

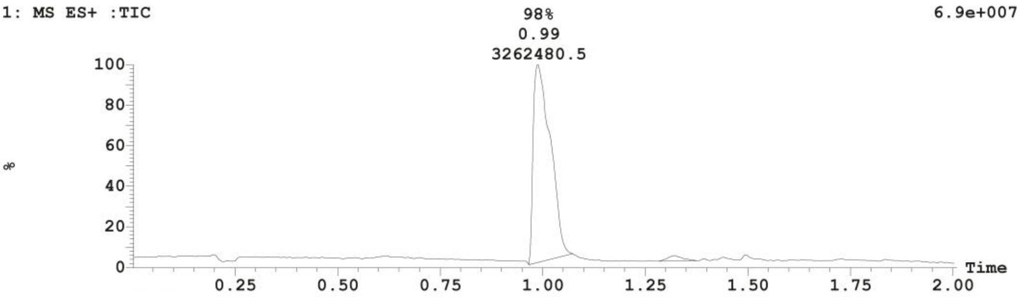

Time	Width	Height	AreaAbs	Area %Total
0.99	0.107	7e+007	2625e+006	97.62
1.32	0.100	2e+006	9516e+004	2.38

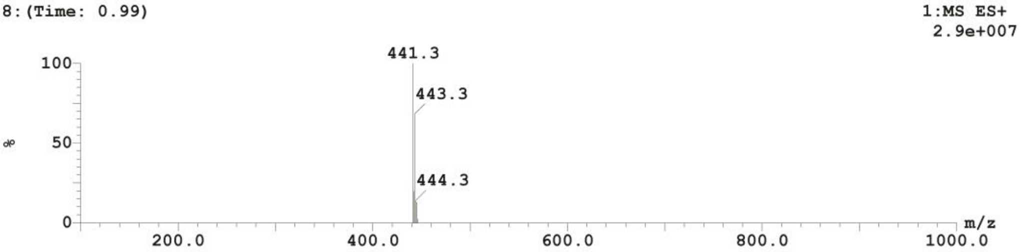

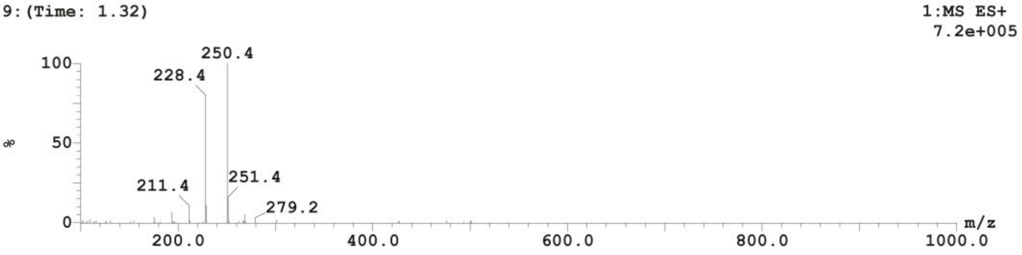

Supplemental Figure 10: LCMS report for compound PRT382

A) LCMS (m/z): [M]+ calculated for $C_{18}H_{18}Cl_2N_4O_5$, 441.3; found, 443.3.

Supplemental Figure 11

a

===

Seq. Line : 3
Acq. Instrument : HPLC003
Location : Vial 53
Inj : 1
Inj Volume : 0.0 µl
Actual Inj Volume : 3.0 µl

Analysis Method : D:\METHODS\WATERS SUNFIRE C18-5MM-4.6-150MM COLUMN\M-AB.M

Method Info : Waters Sunfire C18 5µm, 4.6x150mm Column, 25C, 1.000ml/min
A: 0.03%TFA in H2O, B: 0.03%TFA in ACN

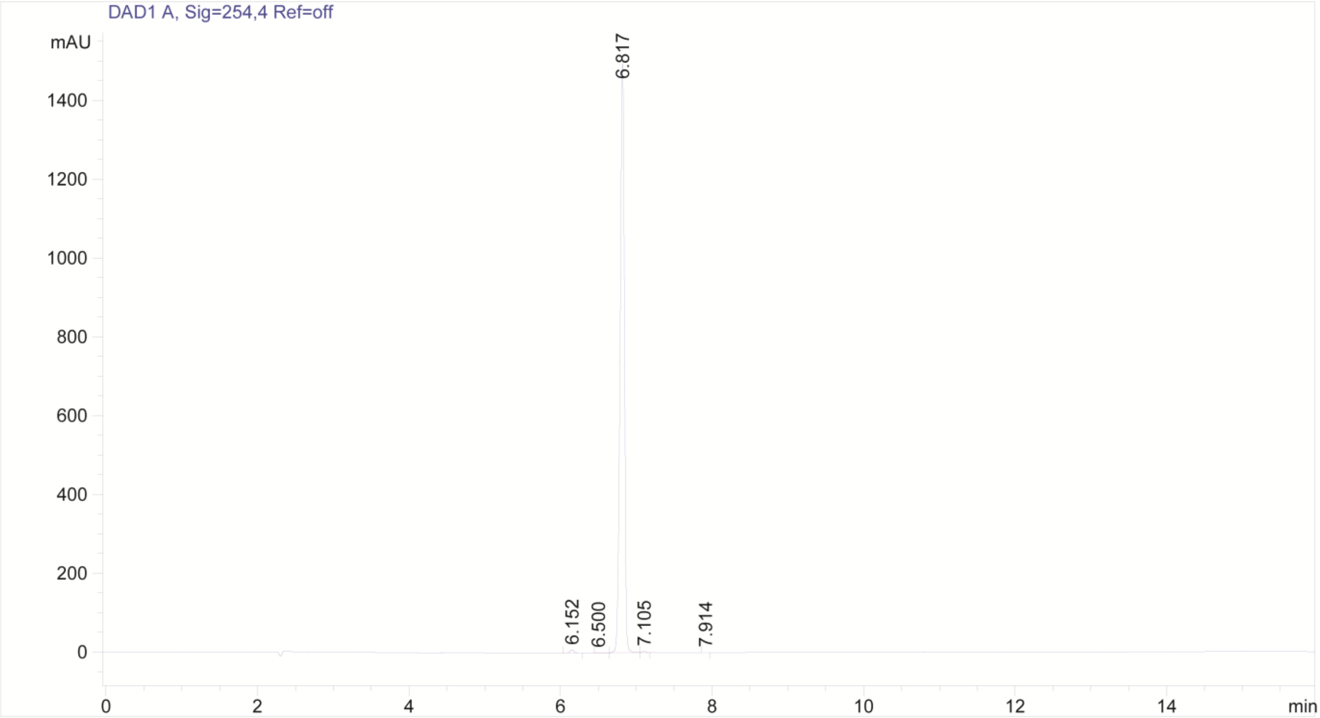

===

Area Percent Report

===

Sorted By : Signal
Multiplier : 1.0000
Dilution : 1.0000
Use Multiplier & Dilution Factor with ISTDs

Signal 1: DAD1 A, Sig=254,4 Ref=off

Peak #	RetTime [min]	Type	Width [min]	Area [mAU*s]	Height [mAU]	Area %
1	6.152	BB	0.0514	24.89300	7.76055	0.4181
2	6.500	BB	0.0580	2.50715	6.64857e-1	0.0421
3	6.817	BV	0.0620	5913.22021	1498.71875	99.3138
4	7.105	VB	0.0527	11.58330	3.32387	0.1945
5	7.914	BB	0.0476	1.87348	6.49665e-1	0.0315
Totals :				5954.07715	1511.11769	

===

Supplemental Figure 11: HPLC report for compound PRT382

A) PRT382 HPLC Chromatogram.
