# Supplementary material for: Dysregulation of PRMT5 in chronic lymphocytic leukemia promotes progression with high risk of Richter’s transformation
Source: Nat Commun. 2023 Jan 6;14:97. doi: 10.1038/s41467-022-35778-1 (PMC9823097; doi:10.1038/s41467-022-35778-1)
Supplement: Supplementary file 3 — Reporting Summary [file 41467_2022_35778_MOESM3_ESM.pdf]

nature portfolio

Corresponding author(s): Rosa Lapalombella, PhD

Last updated by author(s): Dec 7, 2022

Reporting Summary

Nature Portfolio wishes to improve the reproducibility of the work that we publish. This form provides structure for consistency and transparency in reporting. For further information on Nature Portfolio policies, see our Editorial Policies and the Editorial Policy Checklist.

Statistics

For all statistical analyses, confirm that the following items are present in the figure legend, table legend, main text, or Methods section.

n/a	Confirmed	
☐	☒	The exact sample size (*n*) for each experimental group/condition, given as a discrete number and unit of measurement
☐	☒	A statement on whether measurements were taken from distinct samples or whether the same sample was measured repeatedly
☐	☒	The statistical test(s) used AND whether they are one- or two-sided<br>*Only common tests should be described solely by name; describe more complex techniques in the Methods section.*
☐	☒	A description of all covariates tested
☐	☒	A description of any assumptions or corrections, such as tests of normality and adjustment for multiple comparisons
☐	☒	A full description of the statistical parameters including central tendency (e.g. means) or other basic estimates (e.g. regression coefficient) AND variation (e.g. standard deviation) or associated estimates of uncertainty (e.g. confidence intervals)
☐	☒	For null hypothesis testing, the test statistic (e.g. *F*, *t*, *r*) with confidence intervals, effect sizes, degrees of freedom and *P* value noted<br>*Give P values as exact values whenever suitable.*
☒	☐	For Bayesian analysis, information on the choice of priors and Markov chain Monte Carlo settings
☐	☒	For hierarchical and complex designs, identification of the appropriate level for tests and full reporting of outcomes
☒	☐	Estimates of effect sizes (e.g. Cohen's *d*, Pearson's *r*), indicating how they were calculated

Our web collection on statistics for biologists contains articles on many of the points above.

Software and code

Policy information about availability of computer code

Data collection	Graphpad Prismv9.2.0, Kaluza v2.0
Data analysis	All flow cytometry optimizations were performed as previously described36 and analyzed with Kaluza v2.0 software (Becton Dickinson).<br><br>RNA-seq: Sequencing libraries were prepared using Illumina TruSeq stranded mRNA library preparation kit and sequenced on HiSeq 4000 instrument targeting approximately 60-80x106 read pairs per sample. The 150 bp paired-end reads were trimmed for adaptors and low-quality bases with Cutadapt and mapped to the mouse reference genome GRCm38 with STAR aligner v2.6.065. The featureCounts utility66 from the subread package (v1.4.6) was used to generate gene counts based on Ensembl gene annotation release 9567. Pairwise comparisons were performed to identify DEGs using the DESeq2 package (v1.24.0)68.<br><br>Low-input RNA-seq: The Clontech SMARTer v4 kit (Takara Bio USA, Inc.) was used for global preamplification. Illumina sequencing libraries were derived from the resultant cDNA using the Illumina Nextera XT DNA Library Prep Kit following manufacturer's instructions. Libraries were sequenced using an Illumina HiSeq 4000 sequencer paired-end 150 bp protocol to approximately 12 million passed filter clusters per sample. Data processing was performed according to the CLEAR workflow, which identifies reliably quantifiable transcripts in low-input RNA-seq for differentially expressed gene (DEG) transcripts using gene coverage profiles. BCR reads were binned by specific heavy (H) and light-chain V genes. First, we quantified the percentage of reads with heavy, kappa, and lambda chains compared to the number of total sequencing reads. MiXCR (v3.0.5) was used with default parameters to identify preprocessed reads containing CDR3 regions from B-cell heavy, kappa, and lambda chains, generating a list of unique CDR3 sequences associated with their relative abundances and specific V(D)J gene usage. Global DEG analysis was performed using DESeq2 (v1.20.0).<br><br>ATAC-seq and data processing: Libraries were sequenced by the Biomedical Sequencing Facility at CeMM using the Illumina HiSeq3000/4000 platform and the 25-bp paired-end configuration. The 150-nt paired end sequence reads were mapped to the human reference genome (GRCh37/hg19) using the Bowtie2 algorithm with maximum fragment length of 1000 and no-mixed mode. After removal of duplicate reads,

DNase2hotspots software package (https://sourceforge.net/projects/dnase2hotspots) was used to identify hotspots (enriched regions) from ATAC-seq. Consensus hotspots from all the treatment condition for each patient were generated as the union of the overlapping hotspots using R GenomicRanges package, then annotated using R ChIPpeakAnno package.

Circularized Chromosome Conformation Capture (4C): The library was subjected to next-generation sequencing on the Illumina platform. The sequencing reads were analyzed with the R.4Cker pipeline and visualized with the RCircos package mapped to the UCSC.HG19.Human.CytoBandIdeogram (http://genome.ucsc.edu).

Single-cell library preparation and sequencing: For mouse scRNA-seq, libraries were prepared using the Chromium Single Cell 3′ Reagent Kits (v2): Single Cell 3′ Library & Gel Bead Kit v2 (PN-120237), Single Cell 3′ Chip Kit v2 (PN-120236) and i7 Multiplex Kit (PN-120262) (10x Genomics). Murine libraries were sequenced on an Illumina Nextseq 500 to achieve 75bp reads. Optimal cDNA amplicon size was achieved using Covaris machine prior to library construction. The P7 and R2 primers were added during the GEM incubation and the P5, and R1 during library construction via end repair, A-tailing, adapter ligation and PCR. The final libraries contain the P5 and P7 primers used in Illumina bridge amplification. Following the Single Cell 3′ Reagent Kits (v2) user guide (manual part no. CG00052 Rev A.), data was processed by the Cell Ranger pipeline (v2.0.1; 10x Genomics). Count matrices were analyzed using the Seurat package (v2.2.1) in R. Read counts were normalized to library size, scaled by 10,000, log-transformed, and filtered.
Human scRNA-seq libraries were prepared using the Chromium Next GEM Single Cell 5' Kit (v2) (PN-1000263): Library Construction kit (PN-1000190), Chromium Next GEM Chip K Single Cell Kit (PN-1000286), Chromium Single Cell Human BCR Amplification Kit (PN-1000253), and Dual Index Kit TT Set A (PN-1000215) (10x Genomics). Human libraries were sequenced on a NovaSeq 6000 with 300 cycles and paired 150 bp reads and resulted in a minimum of >10,000 reads per cell. The data was processed by the Cell Ranger pipeline (v1.0.1; 10x Genomics). Quality control removed low quality cells with a high percentage of reads mapped to mitochondrial genes (mito counts >2 median absolute deviations above the median) and/or low numbers of genes detected (feature counts >2 median absolute deviations below the median) using functions from blaseRtools (https://github.com/blaserlab/blaseRtools). The Doubletfinder package was used to remove high-likelihood doublets. UMAP dimensionality reduction, clustering, and top marker analysis was achieved using functions from Monocle3. Leiden clustering and k-Means10 clustering was utilized for human and murine analyses, respectively. BCR V(D)J single-cell transcriptome analysis was analyzed using blaseRtools R package. Gene expression profiles for stated clusters (Figure 1H) were aggregated for pseudobulk differential expression analysis using Monocle3 and DESeq2 functions. UMAP plots were generated utilizing blaseRtools. Aggregate gene expression UMAP plots utilized the sum of the normalized per-cell UMI counts for each gene set to generate the aggregate gene expression score.

Statistical analysis: All analyses were performed by the OSU Center for Biostatistics using previously described models using SAS/STAT software, v9.4 of the SAS System for Windows (SAS Institute Inc., Cary, NC).

For manuscripts utilizing custom algorithms or software that are central to the research but not yet described in published literature, software must be made available to editors and reviewers. We strongly encourage code deposition in a community repository (e.g. GitHub). See the Nature Portfolio guidelines for submitting code & software for further information.

Data

Policy information about availability of data

All manuscripts must include a data availability statement. This statement should provide the following information, where applicable:

- Accession codes, unique identifiers, or web links for publicly available datasets
- A description of any restrictions on data availability
- For clinical datasets or third party data, please ensure that the statement adheres to our policy

The RNA-sequencing, scRNA-seq, and ATAC-seq data generated in this study have been deposited in the GEO database under accession code #GSE183432 https://www.ncbi.nlm.nih.gov/geo/query/acc.cgi?acc=GSE183432. Source data are provided with this paper. PRT382 is a preclinical tool compound that it is not commercially available but can be obtained from OSU or Prelude therapeutics upon request. CLL and RT gene expression sets derived from Nadeu and colleagues is available at https://doi.org:10.1038/s41591-022-01927-8 in Supplemental Table 11b.The GRCm38 mouse reference genome is available at (https://www.ncbi.nlm.nih.gov/assembly/GCF_000001635.20/). The UCSC.HG19.Human.CytoBandIdeogram is available at https://rdrr.io/cran/RCircos/man/UCSC.HG19.Human.CytoBandIdeogram.html. ScRNA-seq analysis scripts are provided at https://github.com/blaserlab/lapalombella_whipp. A companion R data package is available at https://doi.org/10.5281/zenodo.7406465.

Field-specific reporting

Please select the one below that is the best fit for your research. If you are not sure, read the appropriate sections before making your selection.

☒ Life sciences ☐ Behavioural & social sciences ☐ Ecological, evolutionary & environmental sciences

For a reference copy of the document with all sections, see nature.com/documents/nr-reporting-summary-flat.pdf

Life sciences study design

All studies must disclose on these points even when the disclosure is negative.

Sample size	For scRNA-seq no statistical methods were used since all patients with material available were included.<br><br>For Eμ-PRMT5 mouse colonies, prior consultation with The Ohio State University Center for Biostatistics determined a samples size of n=40 was sufficient to provide over 80% power to detect a 20% difference in overall mortality between groups, using a two-sided chi-square test with an α=0.05 level of significance. Overall n=75 Eμ-PRMT5 were followed, and n=26 Eμ-PRMT5 mice were chosen for characterization against n=35 Eμ-TCL1 mice and n=10 wildtype littermate mice.

	Histopathology images and flow cytometry plots are representative of experiments with sample size n≥3 per group. Sample size of n≥3 per group were deemed sufficient following assessment of the overall phenotype observed between mouse colonies. This same rationale was used to determine n≥2 per group for RNA-sequencing experiments and n≥4 per group for scRNA-seq experiments. For engraftment and therapeutic studies, similar rationale determined a sample size of n=7 was sufficient to detect response between groups.
Data exclusions	Pre-established protocols excluded from analysis any mice that perished or met removal criteria due to severe fighting wounds or malocclusion.
Replication	Tissue microarray images represent tests from 70 CLL patients and 15 RT patients and were verified by independent pathologists. Eμ-PRMT5 mice from three independent founder lines were followed over several generations for characterization and all replication attempts to verify experimental findings, including engraftment and therapeutic studies, amongst animal models were successful.
Randomization	Organs for histopathology analysis were harvested from Eμ-PRMT5 mice meeting predefined euthanasia criteria and representative tissue samples were randomly selected from a pool of mice having evidence of disease. In engraftment studies, mice were randomly assigned to treatment groups according to the data which their circulating leukemia exceeded the 10% threshold and evenly distributed according to disease severity at that date.<br><br>Not relevant for the human scRNA-seq since no experimental groups or conditions were evaluated.
Blinding	Tissue microarray analysis was conducted by independent expert pathologists prior to initiation of this study and were thus not aware of study goals.<br><br>Stained sections were assessed by veterinary anatomic pathologists blinded to transgenic strain and/or treatment group. In sequencing studies, investigators were blinded to group allocation and study endpoints. Veterinary mouse technicians determining euthanasia criteria were blinded to transgenic status, treatment enrollment group, and disease status. For therapeutic studies, investigators determining group allocation were blinded to therapeutic received by each group.

Reporting for specific materials, systems and methods

We require information from authors about some types of materials, experimental systems and methods used in many studies. Here, indicate whether each material, system or method listed is relevant to your study. If you are not sure if a list item applies to your research, read the appropriate section before selecting a response.

Materials & experimental systems

n/a	Involved in the study	
☐	☒	Antibodies
☐	☒	Eukaryotic cell lines
☒	☐	Palaeontology and archaeology
☐	☒	Animals and other organisms
☐	☒	Human research participants
☒	☐	Clinical data
☒	☐	Dual use research of concern

Methods

n/a	Involved in the study	
☒	☐	ChIP-seq
☐	☒	Flow cytometry
☒	☐	MRI-based neuroimaging

Antibodies

Antibodies used	Antibodies used for immunoblots included:<br>anti-human PRMT5 (1:1000 dilution; Abcam #ab109451)<br>GAPDH (1:10000 dilution; Millipore Sigma #MAB374, clone 6C5)<br>SDMA (1:1000 dilution; Cell Signaling Technology #13222)<br>c-Myc (1:2000 dilution; Cell Signaling Technology #5605S)<br>β-Actin (1:10000 dilution ;Cell Signaling Technology #4967S)<br><br>Immunophenotyping of tumor cells in peripheral blood and spleen of Eμ-PRMT5, Eμ-PRMT5/TCL1 and Eμ-TCL1 mice by flow cytometry was performed as follows:<br>APC rat anti-mouse CD45 (1/50 dilution; BD Biosciences, Cat #559864),<br>FITC rat anti-mouse CD45R/B220 (1/25 dilution; BD Biosciences Cat #553088),<br>BV421 rat anti-mouse CD19 (1/25 dilution; BD Biosciences Cat #562701),<br>PE rat anti-mouse CD5 (1/50 dilution; BD Biosciences Cat #553023),<br>BV510 hamster anti-mouse CD3e (1/150 dilution; BD Biosciences Cat #563024),<br>BV650 rat anti-mouse CD11-b (1/200 dilution; BD Biosciences Cat #653402),<br>BB515 rat anti-mouse CD19 (1/200 dilution; BD Biosciences Cat #564509),<br>BUV737 rat anti-mouse CD5 (1/150 dilution; BD Biosciences Cat #612809).
Validation	Tissue Microarray was done according to the manufacturer's recommendations. Flow cytometry gating strategies followed published data and technical resource publications and were adapted to allow exclusion and interrogation of CD19+CD5+ CLL-like populations. Fluorescence-minus-one (FMO) controls were used for each marker and gate position. Cells were gated on viable single mononuclear

cells.

No customized antibodies were used.

All antibodies used for western blotting and flow cytometry are from commercial sources (Abcam, Millipore Sigma, Cell Signaling Technology, BD Biosciences). Validation data are available on the manufacturer's website and datasheets:

anti-human PRMT5 (Abcam #ab109451) → https://www.abcam.com/prmt5-antibody-epr5772-ab109451.html

GAPDH (Millipore Sigma #MAB374) → https://www.sigmaaldrich.com/US/en/product/mm/mab374

SDMA (Cell Signaling Technology #13222) → https://www.cellsignal.com/products/primary-antibodies/symmetric-di-methyl-arginine-motif-sdme-rg-multimab-rabbit-mab-mix/13222

c-Myc (Cell Signaling Technology #5605S) → https://www.cellsignal.com/products/primary-antibodies/c-myc-d84c12-rabbit-mab/5605?site-search-type=Products&N=4294956287&Ntt=%235605s%29&fromPage=plp&_requestid=300423

β-Actin (Cell Signaling Technology #4967S) → https://www.cellsignal.com/products/primary-antibodies/b-actin-antibody/4967?site-search-type=Products&N=4294956287&Ntt=%234967s%29&fromPage=plp&_requestid=300456

APC rat anti-mouse CD45 (BD Biosciences Cat #559864) → https://www.bdbiosciences.com/en-us/products/reagents/flow-cytometry-reagents/research-reagents/single-color-antibodies-ruo/apc-rat-anti-mouse-cd45.559864

FITC rat anti-mouse CD45R/B220 (BD Biosciences Cat #553088) → https://www.bdbiosciences.com/en-us/products/reagents/flow-cytometry-reagents/research-reagents/single-color-antibodies-ruo/fitc-rat-anti-mouse-cd45r-b220.553088

BV421 rat anti-mouse CD19 (BD Biosciences Cat #562701) → https://www.bdbiosciences.com/en-us/products/reagents/flow-cytometry-reagents/research-reagents/single-color-antibodies-ruo/bv421-rat-anti-mouse-cd19.562701

PE rat anti-mouse CD5 (BD Biosciences Cat #553023) → https://www.bdbiosciences.com/en-us/products/reagents/flow-cytometry-reagents/research-reagents/single-color-antibodies-ruo/pe-rat-anti-mouse-cd5.553023

BV510 hamster anti-mouse CD3e (BD Biosciences Cat #563024)& https://www.bdbiosciences.com/en-us/products/reagents/
flowcytometry-
reagents/research-reagents/single-color-antibodies-ruo/bv510-hamster-anti-mouse-cd3e.563024

BV650 rat anti-mouse CD11b (BD Biosciences Cat #563402)&https://www.bdbiosciences.com/en-us/products/reagents/
flowcytometry-
reagents/research-reagents/single-color-antibodies-ruo/bv650-rat-anti-cd11b.563402

BB515 rat anti-mouse CD19 (BD Biosciences Cat #564509)&https://www.bdbiosciences.com/en-us/products/reagents/
flowcytometry-
reagents/research-reagents/single-color-antibodies-ruo/bb515-rat-anti-mouse-cd19.564509

BUV737 rat anti-mouse CD5 (BD Biosciences Cat #612809)&https://www.bdbiosciences.com/en-nz/products/reagents/
flowcytometry-
reagents/research-reagents/single-color-antibodies-ruo/buv737-rat-anti-mouse-cd5.612809

Eukaryotic cell lines

Policy information about cell lines

Cell line source(s)	MEC-1 and HG3 CLL cell lines were obtained from DSMZ (Braunschweig, Germany). Lymphoma cell lines JVM-2, U-2932, RL, KARPAS-422, Jurkat, Jeko, Pfeiffer, SUDHL were obtained from commercial sources (DSMZ and ATCC [Manassas, Virginia, USA]). CCMCL-1 was provided by Dr. Selina Chen-Kiang from the Department of Pathology and Laboratory Medicine, Weill Cornell Medicine.
Authentication	MEC-1 and HG3 cell identity was reconfirmed via fluorescence in situ hybridization (FISH) using CLL probe panel, i.e. CEP12/13q14/13q34, ATM/p53, BCL6, MYC, IGH/CCND1 (Abbott Molecular, Des Plaines, IL), and 6q21 (Kreatech, Buffalo Grove, IL) (results for 200 analyzed cells were reported). All cell lines were authenticated by short tandem repeat DNA profiling.
Mycoplasma contamination	All cell lines were confirmed to be mycoplasma negative using the MycoAlert™ Mycoplasma Detection Kit from Lonza (Rockland, ME) according to manufacturer instructions and used within 3-4 weeks from thawing.
Commonly misidentified lines (See ICLAC register)	Jurkat and Pfeiffer

Animals and other organisms

Policy information about studies involving animals; ARRIVE guidelines recommended for reporting animal research

Laboratory animals	Transgenic Eμ-PRMT5 mice were generated on a C57BL/6J background at The Ohio State University Comprehensive Cancer Center's Transgenic Mouse Facility. Mice were housed in an environment with 12-hour light/12-hour dark cycle and temperatures were maintained at 75°F +/- 2°F with 30-60% humidity. Food and water were accessible at all times. Mice were grouped into cages and provided enrichment whenever possible.<br><br>An equal ratio of male and female mice were maintained throughout all analyses. Adoptive transfer studies were conducted using 2 month old immune competent C57BL/6J mice.
Wild animals	This study did not involve wild animals
Field-collected samples	This study did not involve field-collected samples
Ethics oversight	All experiments were carried out under protocols approved by The Ohio State University Institutional Animal Care and Use Committee (IACUC #2010A00000152-R3). Pre-defined euthanasia criteria for mice in all transgenic colonies and murine transplant models included lethargy, impaired motility, splenomegaly, enlarged lymph nodes and/or superficial lymphoid tumors greater than 1.6 cm in diameter (or cumulative diameter of 1.6 cm if multiple tumors), decrease in body weight (>20%), development of tumor masses, ruffled fur, hunched back, failure to nest, and loss of appetite.

Note that full information on the approval of the study protocol must also be provided in the manuscript.

Human research participants

Policy information about studies involving human research participants

Population characteristics	All patients examined had CLL as defined by the 2008 IWCLL criteria. RT was defined by the 2016 WHO Classification. Patient characteristics were collected including age, gender, previous lines of therapy, and CLL vs RT diagnosis status. Covariate-relevant characteristics collected include: age at diagnosis, sex (male/female), diagnosis (CLL or RT), IGHV mutation status (un-mutated/mutated), previous treatment (yes/no).
Recruitment	Peripheral blood or lymph node biopsy from patients with CLL or Richter's Transformation was obtained after written informed consent in accordance with the Declaration of Helsinki and under a protocol approved by the institutional review board (IRB) at The Ohio State University. Participants were not prospectively identified for this study. The population is representative for a tertiary referral center without any identifiable bias.
Ethics oversight	Protocol was approved by the Ohio State University

Note that full information on the approval of the study protocol must also be provided in the manuscript.

Flow Cytometry

Plots

Confirm that:

- [x] The axis labels state the marker and fluorochrome used (e.g. CD4-FITC).
- [x] The axis scales are clearly visible. Include numbers along axes only for bottom left plot of group (a 'group' is an analysis of identical markers).
- [x] All plots are contour plots with outliers or pseudocolor plots.
- [x] A numerical value for number of cells or percentage (with statistics) is provided.

Methodology

Sample preparation	Peripheral blood from Eμ-PRMT5 and Eμ-TCL1 transgenic mice, and wildtype littermates was collected monthly via check punch. Cells from the spleens of Eμ-PRMT5 and Eμ-TCL1 having met predefined euthanasia criteria were processed and isolated using methods previously described. Mouse B cells were isolated from whole spleen suspensions using EasySepTM mouse pan B cell isolation kit (STEMCELL Technologies; Cat #19844).
Instrument	Immunophenotyping was conducted using Beckman Coulter Gallios 3-laser-10-color (B5-R3-V2) cell analyzer.
Software	All flow cytometry data were analyzed using KALUZA v2.0 software (Becton Dickinson).
Cell population abundance	Typically labeled 5x10^5 - 1x10^7 cells.

Gating strategy

Gating strategies followed published data and technical resource publications and were adapted to allow exclusion and interrogation of CD19+CD5+ CLL-like populations. Initial gating on whole mouse blood used FSC/SSC to include cell populations and exclude debris. Single cell populations were then gated using FSC/FSC to exclude irregular shaped cells or doublets. Single cells were then gated on count/CD45+ to include lymphocyte populations for further interrogation.

☒ Tick this box to confirm that a figure exemplifying the gating strategy is provided in the Supplementary Information.
